# Acquired Simple Bone Cyst Associated With Lumbar Spinal Canal Stenosis Progression: A Case Report

**DOI:** 10.7759/cureus.56795

**Published:** 2024-03-23

**Authors:** Takehiro Makizono, Takayasu Andou, Gohsuke Hattori, Motohiro Morioka, Hisaaki Uchikado

**Affiliations:** 1 Department of Neurosurgery, Kurume University School of Medicine, Kurume, JPN; 2 Department of Neurosurgery, Kurume University, Kurume, JPN; 3 Department of Neurological Surgery, Uchikado Neuro-Spine Clinic, Fukuoka, JPN

**Keywords:** venous obstruction, hypertrophied ligamentum flavum, lumbar lamina, lumbar spinal canal stenosis, simple bone cyst

## Abstract

A simple bone cyst (SBC) in the posterior lumbar bone structure is very rare. Here, we report a case of SBC at the L5 lumbar lamina with venous obstruction associated with ligamentum flavum thickening. A 59-year-old woman presented with intermittent claudication due to low back pain and bilateral sciatica. A lumbar MRI showed L4-5 lumbar spinal canal stenosis and a T2-weighted image hyperintense lesion in the L5 lamina. Imaging four years earlier showed no lesions in the L5 lamina. Her symptoms improved after lumbar decompression surgery. The L5 lamina lesion was SBC, leading to a diagnosis of venous infarction. The involvement of neovascularization in the mechanism of degenerative hypertrophy in the ligamentum flavum was suggested. In this case, increased venous perfusion and venous obstruction were involved in the formation of the bone cyst.

## Introduction

Lumbar spinal canal stenosis (LCS) is the most common spinal disorder in elderly patients. Hypertrophied ligamentum flavum (HLF) is a major contributor to LCS [1], which may cause compression of the nerve root or cauda equina. In HLF, elastic fibers are replaced by excessive collagen tissue, resulting in fibrosis [2]. Here, we report a very rare case in which the first appearance of a simple bone cyst (SBC) in the lumbar lamina was observed during the exacerbation of LCS symptoms.

## Case presentation

**Figure 1:**
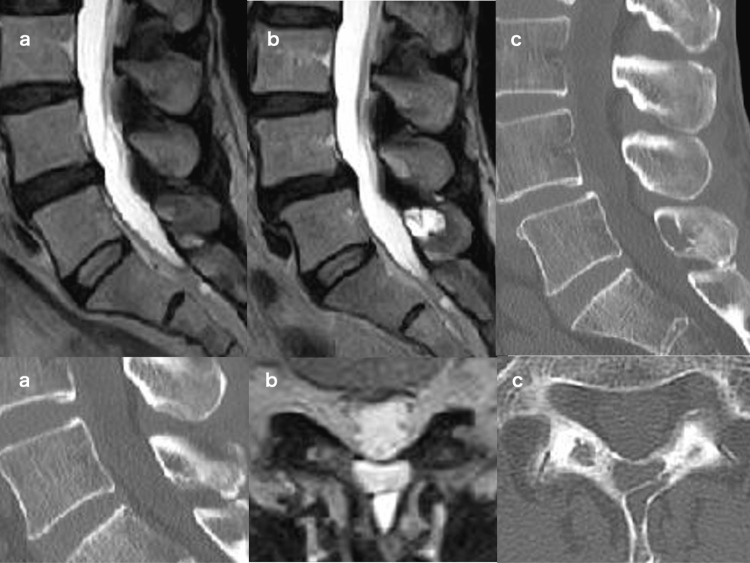
Changes in lumbar spinal images (a) Initial lumbar MRI (upper) and 3D-CT (lower) showed no more obvious findings. (b) MRI at symptom exacerbation showed L4-5 stenosis and spondylolisthesis with HLF and L5 laminar T2WI hyperintensity. Sagittal plane (upper), axial plane (lower). (c) 3D-CT at symptom exacerbation showed intramedullary low density and cavitation in the L5 laminar. Sagittal plane (upper), (lower) MRI: magnetic resonance imaging; CT: computed tomography; HLF: hypertrophied ligamentum flavum; 3D: three dimensional

A 55-year-old woman presented with low back pain. Lumbar MRI and three-dimensional CT (3D-CT) examinations showed no obvious findings (Figure 1a). Follow-up was performed with conservative treatment. Four years later, at age 59, she gradually developed lower back pain and gradually developed bilateral sciatica and intermittent claudication. MRI showed progression of L4-5 canal stenosis and degenerative spondylolisthesis with HLF and intraosseous hyperintensity lesion of T2 weighted image (T2WI) at L5 laminar (Figure 1b). Contrast-enhanced MRI showed no enhanced lesion. Furthermore, 3D-CT revealed a hypo-density lesion at a cancellous bone in the L5 lamina (Figure 1c).

**Figure 2:**
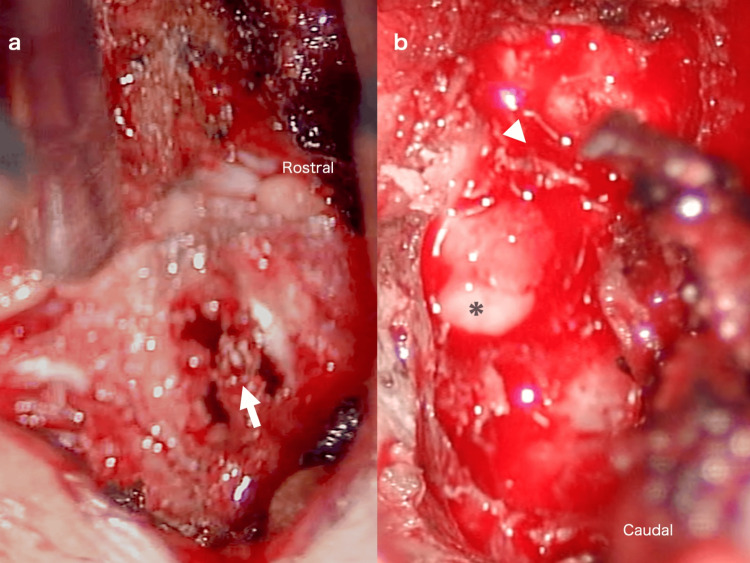
Intraoperative findings (a) Intraoperative findings showed cavitary degeneration of the L5 lamina arch with a bone cyst (arrow), and no tumor cells were found pathologically. (b) After decompressive laminotomy, the epidural venous plexus (asterisk) under the L5 lamina was enlarged with thickened ligamentum flavum at the L4-5 level (arrowhead)

A decompression surgery was performed, and her symptoms improved. Intraoperative findings showed cyst cavitary degeneration of the L5 lamina (Figure 2a), and neovascularization into the thickened ligamentum flavum and enlargement of the epidural venous plexus were observed (Figure 2b). Pathological examination showed an SBC in the lamina bone without obvious neoplastic lesions or HLF.

## Discussion

Unicameral bone cysts, also known as SBC, are common benign non-neoplastic lucent bony lesions that are seen mainly in childhood and typically remain asymptomatic [3]. The etiology and pathogenesis are unknown [3].

Without fracture, the cysts contained clear serosanguineous fluid surrounded by a thin fibrous membranous lining, but this was not our case, and venous obstruction occurred after four years. Obstruction of venous drainage has been suggested to be involved in the formation of SBC [3]. Spinal SBC is very rare and usually occurs in adults. There are few reports of SBC in the lumbar posterior elements [4-6] (Table 1) and vertebrae in middle-aged adults [7].

**Table 1:** Reports of SBC in the lumbar posterior elements SBC: simple bone cyst

Autor	Year	Age	Sex	Level	Part
Chang et al. [4]	2001	25	Male	L5	Lamina
Ha and Kim [5]	2003	53	Female	L1	Pedicle
Ogata et al. [6]	2004	50	Female	L3	Pedicle
Present case	2022	59	Female	L5	Lamina

It is a well-known fact that mechanical stress and angiogenesis are involved in the progression of HLF in LCS [8]. However, the origin from which new blood vessels arise during the progression of HLF is unknown. Anatomically, the most suspect theory is neovascularization from the muscular branch to the multifidus muscles and the posterior vertebral canal artery (prelaminar artery) [9-10]. Their venous return pathway involves the posterior internal venous vertebral plexus within the roof of the spinal canal [9-10]. In this case, these arterial supplies might increase while the venous outlet is obstructed by an unknown cause, resulting in SBC formation. To the best of our knowledge, no reports of SBC of the lumbar lamina caused by venous obstruction have been reported. In this case, it was considered that laminar venous infarction occurred because the prelaminar artery became a feeding vessel during the progression of HLF with posterior epidural venous plexus obstruction. Increased blood flow of HLF from the prelaminar artery can result in venous obstruction, resulting in lamina infarction in this case.

## Conclusions

The involvement of neovascularization in the mechanism of ligamentum flavum degenerative hypertrophy was suggested. Feeding blood vessels to the laminar are prelaminar arteries, and the blood-stealing phenomenon with venous obstruction was shown in this case. The results suggested that SBC may have occurred within the L5 laminar vertebral arch. Acquired SBC associated with venous obstruction in the L5 lamina observed LCS progression.
